# Transcriptomic profiling of autoimmune hepatitis identifies TRAT1 as an *in vitro* negative regulator of NK cell effector functions

**DOI:** 10.3389/fimmu.2026.1843865

**Published:** 2026-07-07

**Authors:** Jiapeng Gao, Lixia Huo, Jianfeng Zhong, Jiexun Cai, Jing Yu, Jingwen Li, Junbo Jiang, Chuanzi Hong, Yunliang Yao, Min Feng

**Affiliations:** 1 First Affiliated Hospital, Huzhou Normal University, Huzhou, China; 2 School of Medicine & Nursing, Huzhou Normal University, Huzhou, China; 3 Huzhou Central Hospital, The Affiliated Central Hospital of Huzhou Normal University, Huzhou, China

**Keywords:** AIH (autoimmune hepatitis), negative regulator, NK cell, PLCγ/Ca2+ pathway, TRAT1

## Abstract

**Background:**

T cell receptor-associated transmembrane adaptor 1 (TRAT1) is a well-characterized regulator of T-cell signaling, yet its functional roles in innate lymphocytes remain largely undefined. This study aimed to identify autoimmune hepatitis (AIH)-associated immune targets and perform an exploratory functional characterization of TRAT1 in NK-cell models.

**Methods:**

We performed transcriptomic analysis on liver tissues from AIH patients and disease controls to prioritize candidate genes. Public datasets and a Concanavalin A (ConA)-induced acute immune-mediated hepatitis murine model were used for contextualization and experimental support for Trat1 expression and immune cell dynamics. The functional role of TRAT1 in NK cells was assessed using NK92-MI cells and primary human NK cells with siRNA-mediated TRAT1 knockdown (KD). TRAT1 expression kinetics and cytotoxicity were examined in both systems, whereas proliferation, effector molecule production, surface receptor expression, calcium flux, and mitogen-activated protein kinase (MAPK) signaling were further analyzed in NK92-MI cells.

**Results:**

TRAT1 was prioritized as an AIH-enriched immune-related candidate gene in this discovery cohort. In the ConA-induced acute immune-mediated hepatitis model, *Trat1* expression was selectively elevated in sorted hepatic NK-cell-enriched populations. *In vitro*, TRAT1 mRNA expression transiently increased in both NK92-MI and primary human NK cells upon activation; its knockdown subsequently enhanced their cytotoxicity against target cells. In NK92-MI cells, TRAT1 deficiency led to enhanced proliferation and elevated production of Interferon-gamma (IFN-γ), Tumor necrosis factor-alpha (TNF-α), Granzyme B (GZMB), and perforin. TRAT1 deficiency also selectively upregulated activating receptors NCR1 (NKp46) and NCR2 (NKp44) while downregulating NKG2D. Mechanistically, TRAT1 KD was associated with increased Ca²^+^influx, phospholipase C gamma 2 (PLCγ2) phosphorylation, and the phosphorylation of ERK1/2 and p38.

**Conclusion:**

Through an exploratory transcriptomic discovery approach, this study suggests that TRAT1 acts as an *in vitro* candidate negative modulator of NK-cell effector functions, potentially involving calcium and MAPK signaling in NK92-MI cells. These data support TRAT1 as a candidate mechanistic lead for future validation in larger clinical cohorts and lineage-specific *in vivo* models.

## Introduction

Autoimmune hepatitis (AIH) is a chronic, progressive liver disease mediated by immune dysregulation, characterized by elevated transaminases, hypergammaglobulinemia, and the presence of autoantibodies (1–3). Despite standard immunosuppressive therapies, a significant proportion of patients respond poorly or experience relapse, leading to end-stage liver disease (2). While the pathogenic roles of adaptive immunity are well established, innate immune cells, including natural killer (NK) cells, are increasingly recognized as potential contributors to hepatic inflammation (4, 5). Therefore, further characterization of the immunological pathways associated with hepatocyte injury may help identify candidate mechanistic leads.

T Cell Receptor Associated Transmembrane Adaptor 1 (TRAT1) primarily functions as a regulator of the T-cell receptor (TCR) and is involved in adaptive immune responses, including Th1, Th2, and Th17 cell differentiation (6, 7). Clinically, TRAT1 has been reported as a potential RNA biomarker for certain malignancies, such as non-small cell lung cancer (6–8). However, while TRAT1 is a known signaling adaptor in T lymphocytes, its expression, functional significance, and mechanistic roles in innate lymphocytes—particularly NK cells—remain largely unexplored.

**Figure 1:**
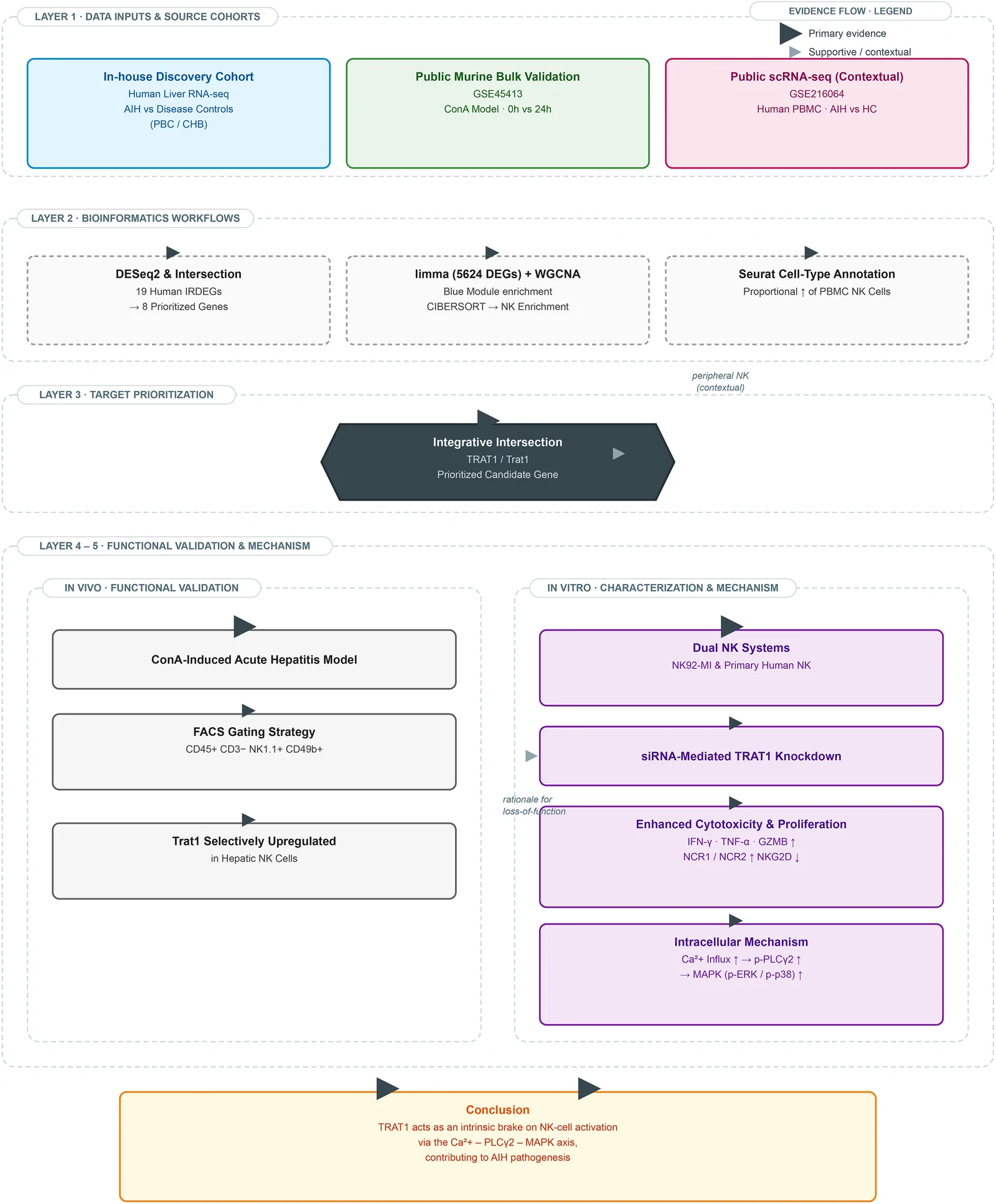
Comprehensive evidence flow and stratified methodological workflow. A schematic outline demonstrating the dynamic integration of clinical data, computational validation, *in vivo* experiments, and *in vitro* characterizations structured into distinct experimental layers. Layer 1 (Data Inputs & Source Cohorts): Delineates the baseline biological resources, including the primary in-house clinical discovery cohort (human liver RNA-seq comparing AIH against disease controls), the public murine bulk validation matrix (GSE45413), and the public single-cell RNA-seq cohort (GSE216064) utilized for cross-verification. Layer 2 (Bioinformatics Workflows): Summarizes the parallel computational pipelines, comprising differential gene selection via DESeq2, modular network generation via WGCNA, and cell-type clustering/annotation via Seurat. Layer 3 (Target Prioritization): Shows the analytical integration and intersection of the three separate data streams, culminating in the prioritization of TRAT1/Trat1 as a prioritized candidate gene of interest. Layer 4 & 5 (Functional Validation & Mechanism): Details the parallel downstream experimental tracks, split between *in vivo* functional confirmation (ConA-induced acute hepatitis murine model combined with subpopulation-specific sorting) and *in vitro* characterizations (siRNA-mediated TRAT1 knockdown across human NK92-MI and primary human NK cell systems), followed by targeted intracellular signaling assays. Conclusion: Represents the conceptual summary linking TRAT1 to the fine-tuning of NK-cell activation through the Ca²^+^–PLCγ2–MAPK signaling axis during immune-mediated hepatic stress.

In this exploratory study, we integrated transcriptomic profiling of liver tissues from patients with AIH, public dataset analyses, an *in vivo* murine hepatitis model, and *in vitro* functional assays (**Figure 1**). This workflow was designed to prioritize candidate immune-related genes and examine the functional directionality of TRAT1 in NK-cell models. Our aim was to determine whether TRAT1 could be prioritized as an AIH-associated candidate regulator of NK-cell responses and to define its functional directionality *in vitro*.

## Materials and methods

### DEGs identification and functional enrichment analysis

For our in-house discovery cohort, raw count matrices were used as the input for the DESeq2 package (v1.44.0) in R software (v4.2.2). Prior to statistical testing, low-expression genes were filtered out (retaining genes with total counts ≥ 10 across all samples). Given the limited sample size of the discovery cohort (n = 3 per group), no genes met the significance threshold after multiple-testing correction. Therefore, for this exploratory candidate-prioritization analysis, differentially expressed genes were provisionally defined using an unadjusted P value < 0.05 and an absolute log2 fold change (|log2FC|) > 1. These results were interpreted as hypothesis-generating signals rather than confirmatory transcriptomic findings. The analysis results were presented by volcano plots and heatmaps using R packages ggplot2 (v3.5.1) and pheatmap (v1.0.12). The clusterProfiler (v4.12.6) R package was utilized to perform functional and pathway enrichment analyses. Gene Ontology (GO) analysis was performed to annotate genes with functions including biological processes (BP), cellular components (CC), and molecular functions (MF). To control the false discovery rate inherent in high-throughput pathway screening, P-values were rigorously adjusted using the Benjamini-Hochberg (BH) multiple-testing correction method. Statistical significance for enriched terms was strictly defined as an adjusted P-value (padj) < 0.05. The Gene Ratio represented in the enrichment plots was mathematically defined as the number of prioritized input genes annotated to a specific Gene Ontology (GO) term divided by the total number of input genes successfully mapped within the GO database.

### Immune-related gene selection and cell infiltration analysis

To identify immune-related genes from the total differentially expressed genes (DEGs), comprehensive reference lists were retrieved from the InnateDB database (downloaded in March 2024) and the ImmPort comprehensive immune gene registry (Release 42). The two independent gene sets were integrated by a union strategy and subsequently deduplicated based on official HGNC human gene symbols, yielding a unified reference catalogue consisting of 3,532 unique immune-related genes. The intersection between this catalogue and our bulk RNA-seq DEGs defined the final human immune-related differentially expressed genes (IRDEGs).

Subsequently, to evaluate the microenvironmental cell composition, immune cell deconvolution of the public murine bulk transcriptomic dataset (GSE45413) was performed using the CIBERSORT (v1.06) algorithm based on the known reference set LM22 (leukocyte signature matrix). Prior to deconvolution, mouse gene symbols from the dataset were mapped and converted to their respective human homologous counterparts utilizing the biomaRt package to bridge the cross-species barrier. The deconvolution analysis was executed with 1,000 permutations. Only samples with a deconvolution P value < 0.05 were retained for downstream exploratory comparisons. The output values are interpreted as estimated immune cell proportions or relative cell fractions rather than absolute quantification.

### Construction and analysis of weighted gene co-expression networks

We constructed a gene co-expression network for immune-mediated hepatitis using the WGCNA (v1.73) R package applied to the public murine dataset (GSE45413). A soft-thresholding power of beta = 6 was selected to ensure a scale-free topology fit index (R^2^) >= 0.85. An adjacency matrix was calculated using a signed Pearson correlation profile, which was subsequently transformed into a topological overlap matrix (TOM). Hierarchical clustering was performed based on TOM-based dissimilarity, and module identification was executed using the Dynamic Tree Cut algorithm with a minimum module size of 30. Highly similar modules were merged utilizing an eigengene correlation coefficient threshold of 0.75, corresponding to a merge cut height of 0.25. Finally, we evaluated the correlation of different modules with the pathogenic status (ConA model vs. control) and selected the most relevant co-expression module for downstream integrative analysis.

### Single-cell RNA-seq analysis and TRAT1-centered module-score analysis

The public PBMC single-cell RNA-seq dataset GSE216064 was processed using Seurat (v5.0.1). Major immune-cell subsets, including T cells, monocytes, NK cells, B cells, and dendritic cells, were annotated based on canonical lineage markers. *TRAT1* transcript distribution was examined across the annotated immune-cell subsets using t-SNE feature plots and violin plots.

To further explore the transcript-level relationship between *TRAT1* and signaling-related gene programs in NK cells, NK cells from the AIH sample were extracted for exploratory *TRAT1*-centered analysis. Pathway signature scores were calculated using the AddModuleScore function in Seurat. This function calculates the average normalized expression of user-defined gene sets in each single cell relative to matched control gene sets, thereby providing transcript-level module scores for selected signaling programs. Gene sets related to Ca²^+^signaling, PLCγ2-associated signaling, ERK/MAPK signaling, and p38/MAPK signaling were evaluated. Spearman correlation analysis was then used to assess the relationship between *TRAT1* expression and these transcript-level module scores in NK cells. Because the dataset contained one AIH sample and one healthy-control sample, all single-cell analyses were interpreted descriptively without inferential statistical testing.

### Induction of ConA-induced acute immune-mediated liver injury in mice

Male C57BL/6 mice were procured from GemPharmatech Co., Ltd. For the study, eight-week-old mice were selected and acclimatized for one week before experimentation. All animal procedures were approved by and performed according to the guidelines of the Institutional Animal Care and Use Committee at Huzhou Central Hospital. To induce acute immune-mediated liver injury, mice in the challenge cohort received a single dose of Concanavalin A (ConA) at 20 mg/kg of body weight, administered via tail vein injection. ConA was prepared by dissolving the lyophilized powder in sterile pyrogen-free phosphate-buffered saline (PBS). Mice in the control cohort were intravenously injected with an equivalent volume of sterile PBS vehicle. At 24 hours post-injection, mice were humanely euthanized to harvest peripheral blood and intact liver tissues for downstream serum transaminase measurement, histopathological assessment, and hepatic mononuclear cell isolation.

Serum alanine aminotransferase (ALT) and aspartate aminotransferase (AST) activities were quantified using the Glutamic-Pyruvic Transaminase/Alanine Aminotransferase (GPT/ALT) Test Kit and the Glutamic Oxaloacetic Transaminase/Aspartate Aminotransferase (GOT/AST) Test Kit, respectively, according to the manufacturer’s instructions (Enzyme-linked Biotechnology, Shanghai, China).

### Animal euthanasia protocol

All animal procedures were approved by the Institutional Animal Care and Use Committee at Huzhou Central Hospital (Approval No. 202112004). Mice were euthanized by carbon dioxide (CO_2_) inhalation in a dedicated chamber. To ensure a humane death and minimize distress, CO2 was introduced at a constant displacement rate of 30% of the chamber volume per minute, strictly adhering to the AVMA Guidelines for the Euthanasia of Animals (2020). Death was confirmed by the sustained absence of respiration and heartbeat for at least one minute.

### Reagents and antibodies

For flow cytometry, antibodies included FITC conjugated anti-mouse CD4 (GK1.5), CD49b(HMα2), anti-human TNF-α (MAb11), NKP46 (9E2), GZMB (QA16A02); PE conjugated anti-mouse NK1.1 (S17016D), anti-human CD56 (QA17A16), NKP30 (P30-15); PE-Cy7 conjugated anti-mouse B220 (RA3-6B2), anti-human NKG2D (1D11); PE-Cy5.5 conjugated anti-mouse CD3 (17A2), APC conjugated anti-mouse CD8a (S18018A), anti-human Perforin (dG9), NKP44 (P44-8), IFN-γ (4S.B3) were purchased from BioLegend (San Diego, CA, USA). For western blot, anti-human TRAT1 (Trim) polyclonal antibody was purchased from Thermo Fisher Scientific (Waltham, Massachusetts, USA); anti-human P38 (D13E1), phospho-P38 (D3F9), ERK1/2 (137F5), phospho-ERK1/2 (D13.14.4E), Plcγ2 (E7L6G) and phospho-Plcγ2 (E9E9Y) were purchased from Cell Signaling Technology (Boston, Massachusetts, USA).

### Flow cytometry

Single-cell suspensions were prepared according to standard protocols. Cells were incubated with antibodies in phosphate-buffered saline (PBS) containing 0.5% fetal bovine serum (FBS) for 30 min at room temperature, washed once with fluorescence-activated cell sorting (FACS) buffer, and analyzed by flow cytometry. For intracellular staining, NK-92MI cells were stimulated with 10 ng/ml phorbol 12-myristate 13-acetate (PMA; Beyotime, Shanghai, China) and 1 μg/ml ionomycin (Beyotime, Shanghai, China) for 2 h at 37 °C in a humidified incubator containing 5% CO_2_. Brefeldin A (10 μg/ml; Beyotime, Shanghai, China) was then added to block intracellular protein transport, and cells were cultured for an additional 4 h. After surface staining, cells were fixed for 20 min with intracellular fixation buffer, permeabilized for 20 min with permeabilization buffer, and subsequently subjected to intracellular staining analysis by flow cytometry. Appropriate isotype control antibodies were included in all experiments to define background staining and distinguish specific binding. Fluorescence-activated cell sorting was performed on a FACSAria Fusion (BD Biosciences, San Jose, CA, USA). Flow cytometric analyses were performed on a FACSCanto™ II (BD Biosciences, San Jose, CA, USA), and data were processed using FlowJo software (Tree Star, Ashland, OR, USA).

### Cell enrichment, culture, and tissue isolation

The NK-92MI cell line, an IL-2-independent derivative, was used to ensure consistent culture conditions. NK-92MI cells were maintained in MEM alpha supplemented with inositol, beta-mercaptoethanol, folic acid, horse serum, fetal bovine serum (FBS), and penicillin-streptomycin (P/S). HepG2 cells were maintained in DMEM supplemented with FBS and P/S. All cell lines were incubated at 37 degrees Celsius with 5% CO2.

For primary cell validation, primary human NK cells were isolated from healthy donor peripheral blood mononuclear cells (PBMCs) via magnetic negative selection using the Human NK Cell Isolation Kit (RWD Life Science) according to the manufacturer’s protocol. Briefly, PBMCs were suspended in chilled separation buffer, labeled with Human NK Biotin Antibody, and conjugated with Streptavidin Magnetic Beads. The mixture was passed through a LarSep Column secured within a magnetic field, and the unlabeled effluent containing enriched primary NK cells was collected. Isolated primary NK cells were cultured in RPMI 1640 supplemented with FBS, P/S, and recombinant human IL-2 for 24 hours before downstream assays.

To isolate murine hepatic mononuclear cells, mice were anesthetized, and the liver was perfused *in situ* via the portal vein with pre-warmed calcium- and magnesium-free Hanks’ Balanced Salt Solution (HBSS) buffer, followed by digestion buffer containing collagenase IV and DNase I. Digested liver tissues were mechanically dissociated and passed through a cell strainer to obtain a single-cell suspension. Hepatic mononuclear cells were further enriched utilizing a Percoll density gradient centrifugation approach. Contaminating erythrocytes were eliminated using red blood cell lysis buffer, and the remaining high-purity hepatic immune cells were resuspended for downstream flow cytometric analysis.

### Real-time quantitative polymerase chain reaction

Total RNA was extracted from cultured cells or mouse immune cells using the EZ-10 Total RNA Mini-Preps Kit (Sangon Biotech, Shanghai, China) according to the manufacturer’s instructions. RNA samples were reverse-transcribed using EasyQuick RT MasterMix (Cwbiotech, Taizhou, Jiangsu, China). Quantitative real-time PCR (qRT-PCR) was performed with UltraSYBR Mixture (Cwbiotech, Taizhou, Jiangsu, China). β-actin was used as the internal reference gene. Relative gene expression was calculated using the 0.5^ΔCt method, where ΔCt = Ct(target gene) − Ct(β-actin). This calculation is equivalent to 2^-^ΔCt.

### Western blot analysis

Cells were harvested, washed with phosphate-buffered saline (PBS), and lysed on ice for 20–30 min in radioimmunoprecipitation assay (RIPA) buffer (Beyotime, Shanghai, China) supplemented with protease and phosphatase inhibitor cocktails (Beyotime, Shanghai, China). Lysates were centrifuged at 4 °C for 10 min, and supernatants were collected for subsequent immunoblot analysis. For each sample, 20 µg of total protein was loaded onto SDS-PAGE gels.

### siRNA transfection

The small interfering RNA (siRNA) targeting human TRAT1 was synthesized by GenePharma (Shanghai, China). The sequences used were as follows: TRAT1 siRNA sense, 5′-CACCAGGGUUGAUGAGUAUTT-3′; antisense, 5′-AUA CUC AUC AAC CCU GGU GTT-3′. NK92-MI cells were transiently transfected using the Amaxa Cell Line Nucleofector Kit R (Lonza, Morristown, NJ, USA). Primary human NK cells were transfected using the Human NK Cell Nucleofector^®^ Solution (Lonza, Morristown, NJ, USA). Nucleofection procedures were performed according to the manufacturer’s instructions. TRAT1 mRNA expression levels were evaluated at 48 hours post-transfection to confirm knockdown efficiency prior to downstream functional assays.

### Cytotoxicity assay

A standard lactate dehydrogenase (LDH) release assay was performed to quantify NK cell-mediated cytotoxicity against target cells. *In vitro* cytotoxicity was assessed using the LDH Cytotoxicity Assay Kit (Beyotime, Shanghai, China) according to the manufacturer’s instructions. Briefly, HepG2 target cells (1 × 10^4^ per well) were seeded in 96-well plates as target cells and incubated with NK92-MI cells or primary human NK cells at indicated effector-to-target (E:T) ratios for the indicated time intervals (3, 6, and 12 h). LDH release reagent (volume corresponding to 10% of medium) was then added into wells designated for maximum LDH release controls, gently mixed, and incubated at 37 °C for 1 h. Plates were subsequently centrifuged at 400 × g for 5 min, and supernatants (60 µl) were transferred to new 96-well plates. LDH detection reagent (60 µl) was added into each well, mixed gently, and incubated for 30 min in the dark. Absorbance values were measured at 490 nm.

### Calcium mobilization assays

NK92-MI cells were incubated with 5 μM Fluo-4 AM (Beyotime, Shanghai, China) in serum-free RPMI 1640 medium for 60 min at 37 °C, and washed twice with calcium-free Hank’s balanced salt solution (HBSS, pH 7.4; Beyotime, Shanghai, China). Following washing, the cells were resuspended in calcium-free HBSS at a density of 3 × 10^6^ cells/ml and incubated at 37 °C for 10 min prior to analysis.

### Clinical information and participants

Research involving human subjects was approved by the Medical Ethics Committee of Huzhou Central Hospital, and informed consent was obtained from all participants. A total of 9 patients were enrolled in this study, comprising 3 patients with Autoimmune Hepatitis (AIH), 3 with Primary Biliary Cholangitis (PBC), and 3 with Chronic Hepatitis B (CHB). The demographic characteristics were as follows: The AIH group consisted of 3 females with a median age of 63 years (range: 52–69 years). The PBC group included 3 females with a median age of 51 years (range: 46–58 years), and the CHB group comprised 1 female and 2 males with a median age of 55 years (range: 45–55 years). All patients met the standard diagnostic criteria for their respective diseases. Specifically, patients diagnosed with AIH met the classification criteria outlined in the Guidelines on the Diagnosis and Management of Autoimmune Hepatitis (9). As controls, patients with CHB and PBC were included according to their respective standard clinical diagnostic guidelines. We utilized residual liver biopsy tissues initially collected through ultrasound-guided percutaneous biopsy using a 16G needle for routine clinical diagnostic purposes. Following collection, tissues were immediately preserved in cold HBSS buffer for subsequent dissociation and sequencing.

### Bulk RNA-seq

Total RNA was isolated using RNeasy mini kit (Qiagen, Germany). Strand-specific libraries were prepared using the TruSeq Stranded mRNA Sample Preparation kit (Illumina, USA) following the manufacturer’s instructions. Briefly, mRNA was enriched with oligo(dT) beads. Following purification, the mRNA was fragmented into small pieces using divalent cations under 86°C for 6 min. The cleaved RNA fragments were copied into first strand cDNA using reverse transcriptase and random primers. This is followed by second strand cDNA synthesis using DNA Polymerase I and RNase H. These cDNA fragments then went through an end repair process, the addition of a single ‘A’ base, and then ligation of the adapters. The products were then purified and enriched with PCR to create the final cDNA library. Purified libraries were quantified by Qubit 2.0 Fluorometer (Life Technologies, USA) and validated by Agilent 2100 bioanalyzer (Agilent Technologies, USA) to confirm the insert size and calculate the mole concentration. Cluster was generated by cBot with the library diluted to 10 pM and then sequenced on the Illumina NovaSeq 6000 (Illumina, USA). The library construction and sequencing were performed at Shanghai Biotechnology Corporation.

### Statistical analysis

Statistical analyses were performed using GraphPad Prism 10 and R software. For basic quantitative data, two-group comparisons were performed using an unpaired two-tailed Student’s t-test. For time-course experiments comparing two groups over multiple time points, data were analyzed using a two-way ANOVA followed by Bonferroni correction for multiple comparisons. Data are presented as mean ± SD unless otherwise indicated. P value < 0.05 was considered statistically significant. Standardized statistical notation is used across all figures: ns, not significant; *P < 0.05; **P < 0.01; ***P < 0.001; ****P < 0.0001. For transcriptomic (DESeq2, limma), enrichment (GO), co-expression (WGCNA), and deconvolution (CIBERSORT) analyses, the specific statistical models and multiple-testing correction methods are detailed in their respective subsections above.

### Software environment and data availability

All bioinformatic analyses and statistical computing were executed within the R software environment (v4.2.2). The specific version controls for the core analytical packages implemented throughout this study were standardized as follows: DESeq2 (v1.44.0) for human differential expression analysis, limma (v3.54.0) for murine microarray screening, WGCNA (v1.73) for weighted gene co-expression network construction, CIBERSORT (v1.06) for immune cell fraction deconvolution, and Seurat (v5.0.1) for single-cell transcriptomic processing.

## Results

### Identification and characterization of immune-related differentially expressed genes in AIH patient livers

To prioritize immune-related candidate genes and organize the subsequent functional analyses, we established an integrative workflow combining human liver transcriptomics, public datasets, an *in vivo* murine hepatitis model, and *in vitro* NK-cell assays (**Figure 1**).

**Figure 2:**
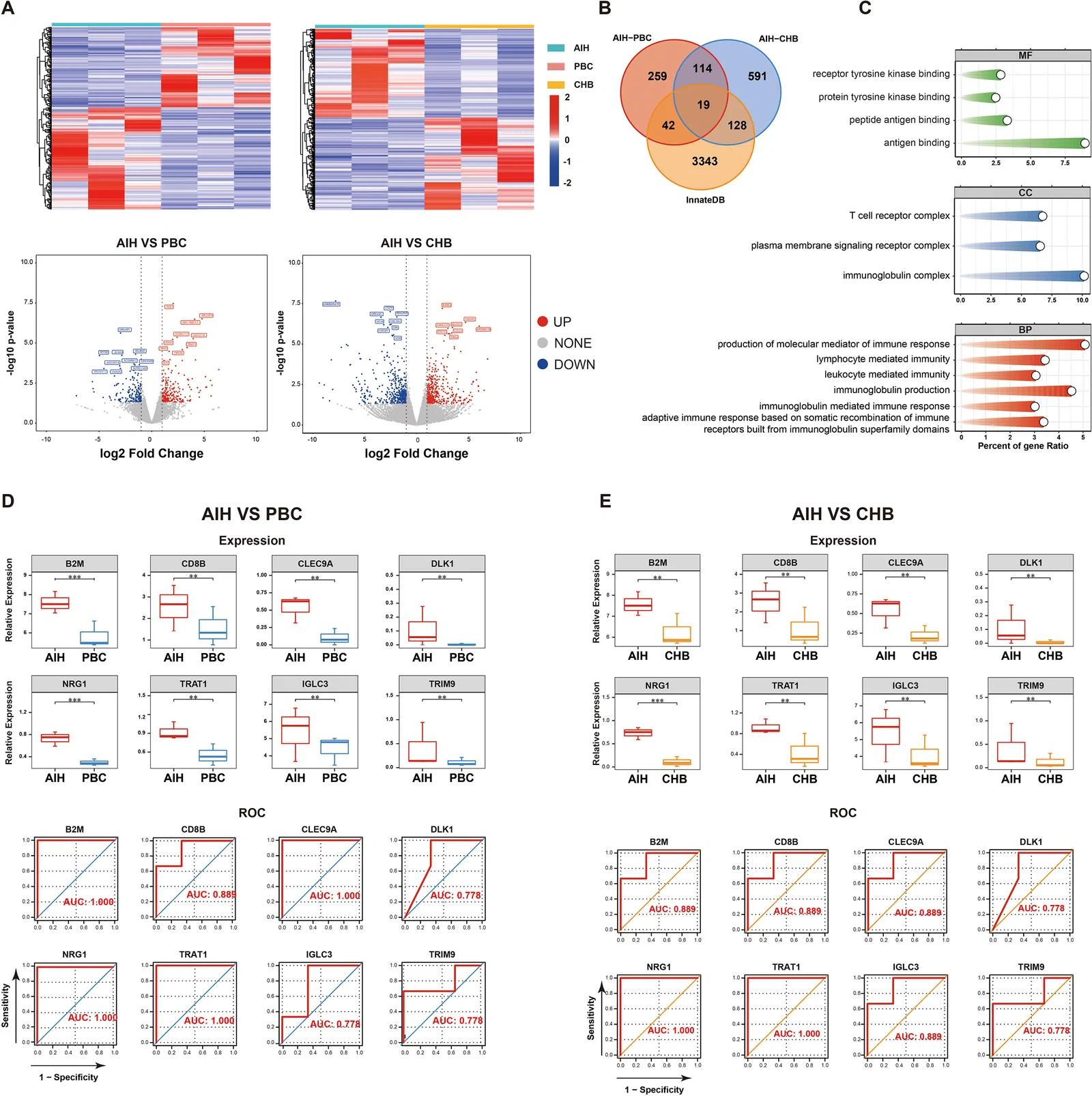
Identification and characterization of immune-related differentially expressed genes (IRDEGs) in AIH. **(A)** Heatmaps (top) and volcano plots (bottom) illustrate differentially expressed genes (DEGs) in liver tissues from AIH patients compared with PBC patients (left panels) and CHB patients (right panels). In volcano plots, red dots represent upregulated genes, blue dots represent downregulated genes, and grey dots represent genes with no significant difference in expression. (|logFC| >1 and p <0.05) **(B)** Venn diagram showing the intersection of DEGs identified from AIH vs. PBC and AIH vs. CHB comparisons with immune-related genes retrieved from the InnateDB and ImmPort databases, yielding 19 common immune-related DEGs (IRDEGs). **(C)** Gene Ontology (GO) enrichment analysis of the 19 IRDEGs. Bar charts display the top enriched terms for Biological Process (BP), Cellular Component (CC), and Molecular Function (MF). The length of the bars corresponds to the -log10(P-value), and the percentage of gene ratio is also indicated. **(D)** Evaluation of candidate discriminatory signals for distinguishing AIH from PBC. Top panels: Boxplots showing the relative expression of eight candidate genes in AIH samples compared to PBC samples. Bottom panels: Corresponding Receiver Operating Characteristic (ROC) curves showing exploratory discriminatory signals for target-prioritization purposes (AUC) of these genes for specifically differentiating AIH from PBC. **(E)** Evaluation of candidate discriminatory signals for distinguishing AIH from CHB. Top panels: Boxplots showing the relative expression of the candidate genes in AIH samples compared to CHB samples. Bottom panels: Corresponding ROC curves for differentiating AIH from CHB. Statistical significance for candidate gene expression comparisons between AIH and disease-control samples was determined using an unpaired two-tailed Student’s t-test. For the exploratory discovery cohort, DEGs were provisionally defined using an unadjusted P value < 0.05 and |log2FC| > 1. GO enrichment analyses were evaluated using Benjamini–Hochberg-adjusted P values as detailed in the Methods. ROC curves are presented solely for exploratory target-prioritization purposes. ns, not significant; *P < 0.05; **P < 0.01; ***P < 0.001; ****P < 0.0001.

As the primary discovery anchor of this matrix, we first performed high-throughput bulk transcriptome sequencing analysis on residual liver aspirate samples from patients diagnosed with AIH, PBC, or CHB. Following standardized data processing and quality control, we identified 434 differentially expressed genes (DEGs; 243 upregulated, 191 downregulated) when comparing AIH with PBC samples, and 850 DEGs (416 upregulated, 434 downregulated) when comparing AIH with CHB samples (**Figure 2A**).

Subsequently, we retrieved 3,532 immune-related genes from the InnateDB and ImmPort databases. Intersection of these genes with the DEGs identified between AIH and the control groups (PBC and CHB) yielded 19 common immune-related DEGs (IRDEGs) (**Figure 2B** and **Supplementary Table 1**). Gene Ontology (GO) enrichment analysis of these IRDEGs revealed that Biological Process (BP) terms were primarily associated with “production of molecular mediators of immune response,” “lymphocyte-mediated immunity,” and “immunoglobulin production.” Cellular Component (CC) analysis showed enrichment in structures such as the “T-cell receptor complex” and “immunoglobulin complex.” Molecular Function (MF) analysis indicated predominant involvement in “antigen binding” and “receptor tyrosine kinase binding” (**Figure 2C**). These enrichment results indicate that the prioritized IRDEGs are associated with immune-response and receptor-signaling pathways.

After excluding immunoglobulin and T-cell receptor variable region genes from the 19 IRDEGs, eight genes remained (marked with asterisks in **Supplementary Table 1**). To evaluate their target-prioritization value, we analyzed both their expression levels and exploratory discriminatory performance in a pairwise manner. As shown in **Figure 2D**, these genes exhibited significantly higher relative expression in AIH samples compared to PBC samples (top panels), and ROC analysis indicated their exploratory discriminatory potential for specifically distinguishing AIH from PBC (bottom panels). Similarly, comparisons between AIH and CHB (**Figure 2E**) demonstrated consistent upregulation of these markers in AIH (top panels), with each gene achieving a calculated area under the curve (AUC) exceeding 0.7 (bottom panels; the bootstrap-estimated 95% confidence intervals are detailed in **Supplementary Table 1**). However, given the limited sample size (n=3 per group) and the absence of healthy liver controls, these observations serve as initial target prioritization rather than definitive biomarker validation.

### Validation of AIH-enriched genes and immune cell subpopulations using public datasets

To further elucidate AIH-enriched genes and immune cell dynamics, we sought validation using publicly available datasets: GSE45413 (transcriptomic data from liver tissue of concanavalin A (ConA)-induced murine model of immune-mediated hepatitis) and GSE216064 (single-cell RNA sequencing, scRNA-seq) data from peripheral blood mononuclear cells (PBMCs) of AIH patients and healthy controls.

**Figure 3:**
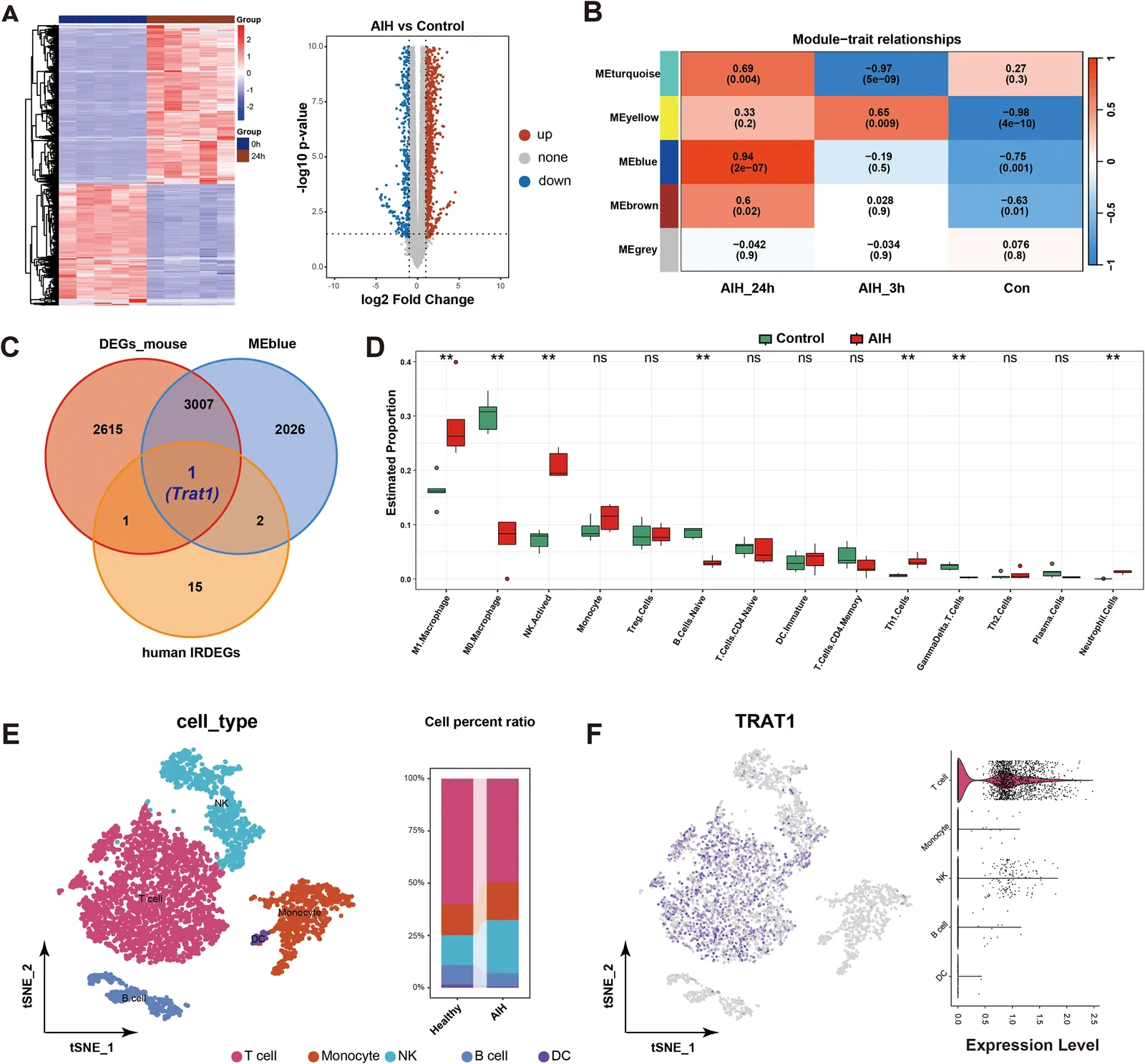
Validation of AIH-enriched genes and immune cell subpopulations using public datasets. **(A)** Heatmap (left) and volcano plot (right) illustrating differentially expressed genes (DEGs) in liver tissue from a concanavalin A (ConA)- induced murine acute immune-mediated hepatitis model (GSE45413) at 24 hours post-injection compared to the 0-hour timepoint. In the volcano plot, red dots represent upregulated genes, blue dots represent downregulated genes, and grey dots represent genes with no significant difference in expression. **(B)** Weighted gene co-expression network analysis (WGCNA) module-trait relationships in the murine ConA-induced hepatitis model. The heatmap shows the correlation between co-expression modules (y-axis) and treatment status (AIH model at different time points vs. control; x-axis). The blue module shows the strongest positive correlation with the ConA-induced hepatitis phenotype at 24 hours. Color intensity and numbers indicate the correlation coefficient and (P-value). **(C)** Venn diagram illustrating the intersection of genes from the WGCNA blue module, murine liver DEGs (from ConA-induced AIH model), and the 19 human immune-related DEGs (IRDEGs) identified in the patient cohort (using murine orthologs). *Trat1* is highlighted as a common prioritized candidate gene. **(D)** CIBERSORT-based deconvolution analysis showing CIBERSORT-inferred estimated proportions of immune-cell populations in liver tissue from ConA-treated and control mice. Significant alterations are noted for Macrophages, activated NK cells, Naive B cells, Th1 cells, γδ T cells, and Neutrophils. ns, not significant; *P < 0.05; **P < 0.01; ***P < 0.001; ****P < 0.0001. **(E)** t-distributed stochastic neighbor embedding (t-SNE) plot visualizing major immune cell types (T cells, monocytes, NK cells, B cells, and dendritic cells (DCs)) identified from single-cell RNA sequencing (scRNA-seq) data of peripheral blood mononuclear cells (PBMCs) from an AIH patient and a healthy control (GSE216064). Cells are colored by annotated cell type. The bar chart inset compares the percentage of these major immune cell types in PBMCs between the AIH patient and the healthy control, showing the largest proportional difference in the NK-cell compartment between the AIH sample and the healthy-control sample. **(F)** Feature plot and violin plot showing *TRAT1* transcript distribution across annotated PBMC immune-cell subsets in GSE216064. Differential expression analysis of the public bulk RNA-seq dataset was performed with multiple-testing correction. Module–trait correlations in WGCNA were assessed by Pearson correlation. For CIBERSORT-inferred estimated immune-cell proportions, group comparisons were performed using an unpaired two-tailed Student’s t-test. Single-cell RNA-seq data are presented as descriptive analyses due to the limited sample size (n=1 per group). ns, not significant; *P < 0.05; **P < 0.01; ***P < 0.001; ****P < 0.0001.

In the murine ConA-induced acute immune-mediated hepatitis dataset (GSE45413), analysis with the limma R package identified 5,624 DEGs in liver tissue from ConA-injected mice at 24 hours post-injection compared to the 0-hour timepoint (**Figure 3A**; **Supplementary Table 2**). Weighted gene co-expression network analysis (WGCNA), using treatment status (AIH model vs. control) as the trait, identified five co-expression modules. The blue module exhibited the strongest positive correlation with the ConA-induced hepatitis phenotype at 24 hours (**Figure 3B**; **Supplementary Table 3**). Intersecting the genes from this blue module, the murine liver DEGs, and the 19 human IRDEGs previously identified in our patient cohort (using murine orthologs for comparison) highlighted *Trat1* as a prioritized immune-related candidate gene for downstream functional evaluation (**Figure 3C**). Furthermore, CIBERSORT-based deconvolution suggested differences in the estimated proportions of several immune-cell populations, including macrophages, activated NK cells, naïve B cells, Th1 cells, γδ T cells, and neutrophils, between ConA-treated and control samples at 24 h (**Figure 3D**).

To investigate corresponding alterations in immune-cell subsets and *TRAT1* expression in human AIH, we analyzed the public PBMC scRNA-seq dataset GSE216064, which includes one AIH sample and one healthy-control sample. Following normalization, dimensionality reduction, clustering, and cell-type annotation using Seurat, five major immune-cell types were identified, including T cells, monocytes, NK cells, B cells, and dendritic cells (DCs). A descriptive comparison between the AIH sample and the healthy-control sample showed that NK cells exhibited the largest proportional difference among the annotated immune-cell populations (**Figure 3E**).

We next examined *TRAT1* mRNA distribution across these immune-cell subsets. *TRAT1* transcripts were detectable predominantly in lymphoid populations, including T cells and NK cells, whereas comparatively lower expression was observed in monocytes, B cells, and DCs (**Figure 3F**). Because this public dataset contained only one AIH sample and one healthy-control sample, these results were interpreted as descriptive transcript-level context rather than inferential evidence of disease-associated expression changes.

To further address whether *TRAT1* expression in NK cells was accompanied by signaling-related transcription patterns, we performed an exploratory *TRAT1-*centered analysis in NK cells from the AIH sample. A transcript-level correlation matrix and module-score analysis were generated for selected Ca²^+^/PLCγ2/MAPK-related genes or gene programs (**Supplementary Figure 2**). No clear transcript-level correlation was observed between *TRAT1* expression and Ca²^+^signaling, PLCγ2-related, ERK/MAPK, or p38/MAPK transcript-level scores.

### Trat1 expression in hepatic immune cells of a ConA-induced acute hepatitis model

**Figure 4:**
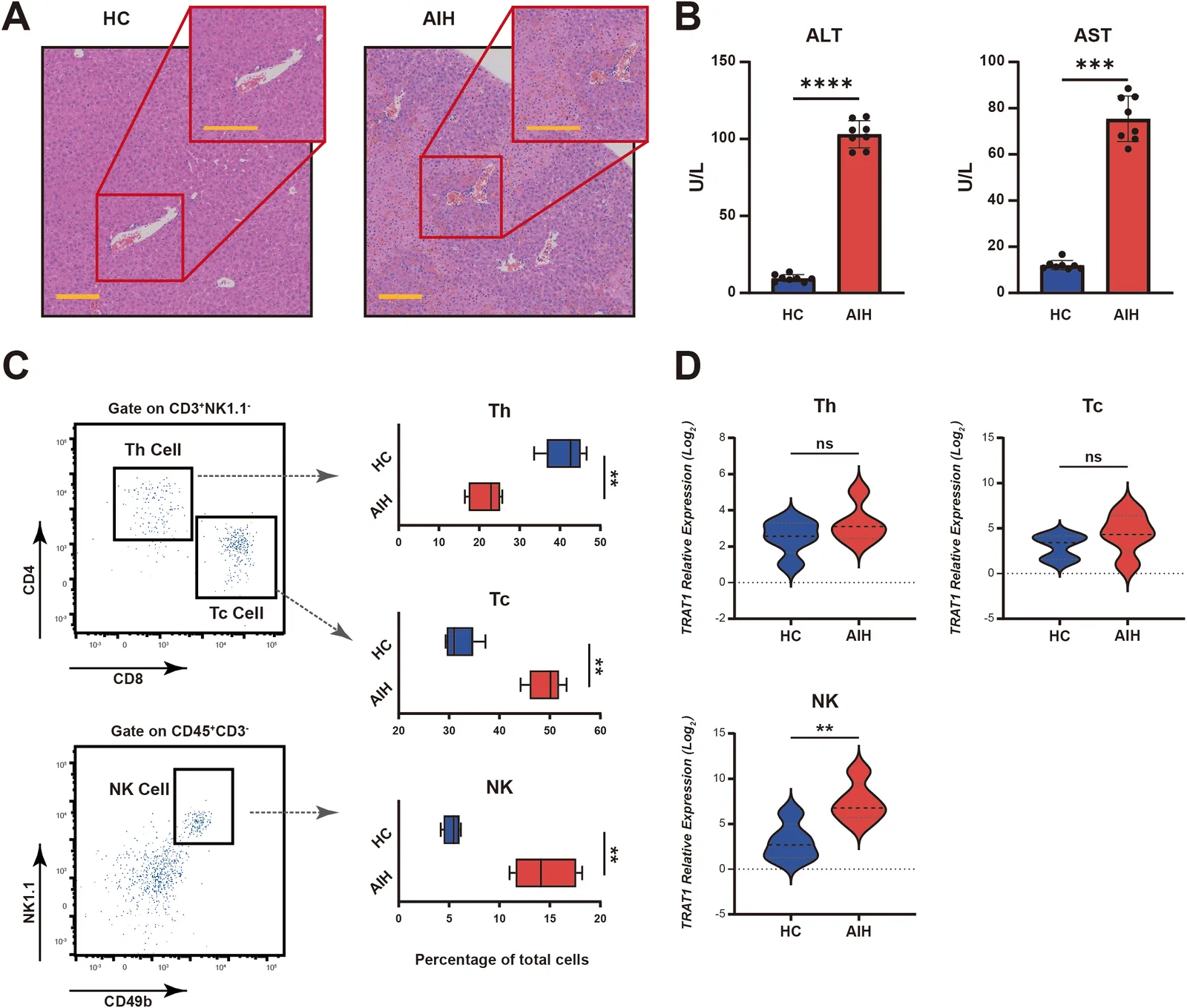
Trat1 expression in hepatic immune cells of a ConA-induced acute hepatitis model. **(A)** Representative H&E stained liver sections from vehicle-treated healthy control (HC) mice and mice with acute hepatitis induced by Concanavalin A (ConA) injection. Red boxes indicate regions selected for higher magnification. High-magnification insets (top right) provide detailed views of the inflammatory infiltration. Scale bars = 100 μm. **(B)** Serological quantification of serum alanine aminotransferase (ALT) and aspartate aminotransferase (AST) levels in HC and ConA-treated mice. **(C)** Flow cytometric analysis of hepatic lymphocyte populations. Representative flow cytometry plots (left) show the gating strategies for Helper T cells (Th; CD3+NK1.1-CD4+CD8-), cytotoxic T cells (Tc; CD3+NK1.1-CD4-CD8+), NK-cell-enriched populations (CD45+CD3-NK1.1+CD49b+ within CD45+CD3- gated cells). Quantitative boxplots (right) display the percentage of these lymphocyte subsets within total liver lymphocytes. **(d)** Relative *Trat1* gene expression in sorted hepatic Th cells, Tc cells, and NK-cell-enriched populations from vehicle-treated and ConA-treated mice, assessed by qRT-PCR and calculated using the 0.5^ΔCt method with β-actin as the internal reference gene. Data represent mean ± SD (n = 6–8 mice per group); statistical significance between groups was determined using an unpaired two-tailed Student’s t-test for each indicated comparison. ns, not significant; *P < 0.05; **P < 0.01; ***P < 0.001; ****P < 0.0001.

To examine the expression pattern of Trat1 *in vivo*, we established a Concanavalin A (ConA)-induced acute immune-mediated hepatitis murine model, which captures acute leukocyte activation and hepatic injury. Histopathological analysis via H&E staining demonstrated that ConA challenge induced liver damage, characterized by inflammatory cell infiltration and focal necrosis around the portal and centrilobular tracks, contrasting with the preserved architecture in vehicle-treated controls (**Figure 4A**). Consistently, serological assessments revealed elevations in both serum ALT and AST levels in the ConA group, confirming acute hepatocyte injury (**Figure 4B**).

To map the cellular distribution of Trat1 during acute hepatic inflammation, we conducted flow cytometric immunophenotyping of hepatic mononuclear cells. Given that previous studies have predominantly characterized TRAT1 as a regulator of T-cell signaling, we focused our immunophenotyping on hepatic CD4+ T cells and CD8+ T cells as classical reference lineages (10), while extending our evaluation to investigate the uncharacterized role of TRAT1 in NK cells. Utilizing a gating strategy designed to separate these specific lymphocyte subsets, we observed elevations in the estimated relative frequencies of hepatic cytotoxic T cells (Tc) and natural killer (NK) cells, along with a drop in helper T cells (Th), within the total intrahepatic lymphocyte pool following ConA administration (**Figure 4C**). Subsequently, fluorescence-activated cell sorting (FACS) combined with qPCR analysis on these isolated subpopulations revealed that while Trat1 expression displayed a non-significant upward trend in Th and Tc cell compartments, its expression was elevated in sorted hepatic NK-cell-enriched populations during the acute injury phase (**Figure 4D**).

As supplementary contextual evidence, a preliminary CYP2D6 plasmid-induced chronic immune-mediated liver injury model showed a similar increase in Trat1 expression in the hepatic NK-cell-enriched population (**Supplementary Figure 1**).

### TRAT1 negatively regulates NK cell activation and cytotoxicity

**Figure 5:**
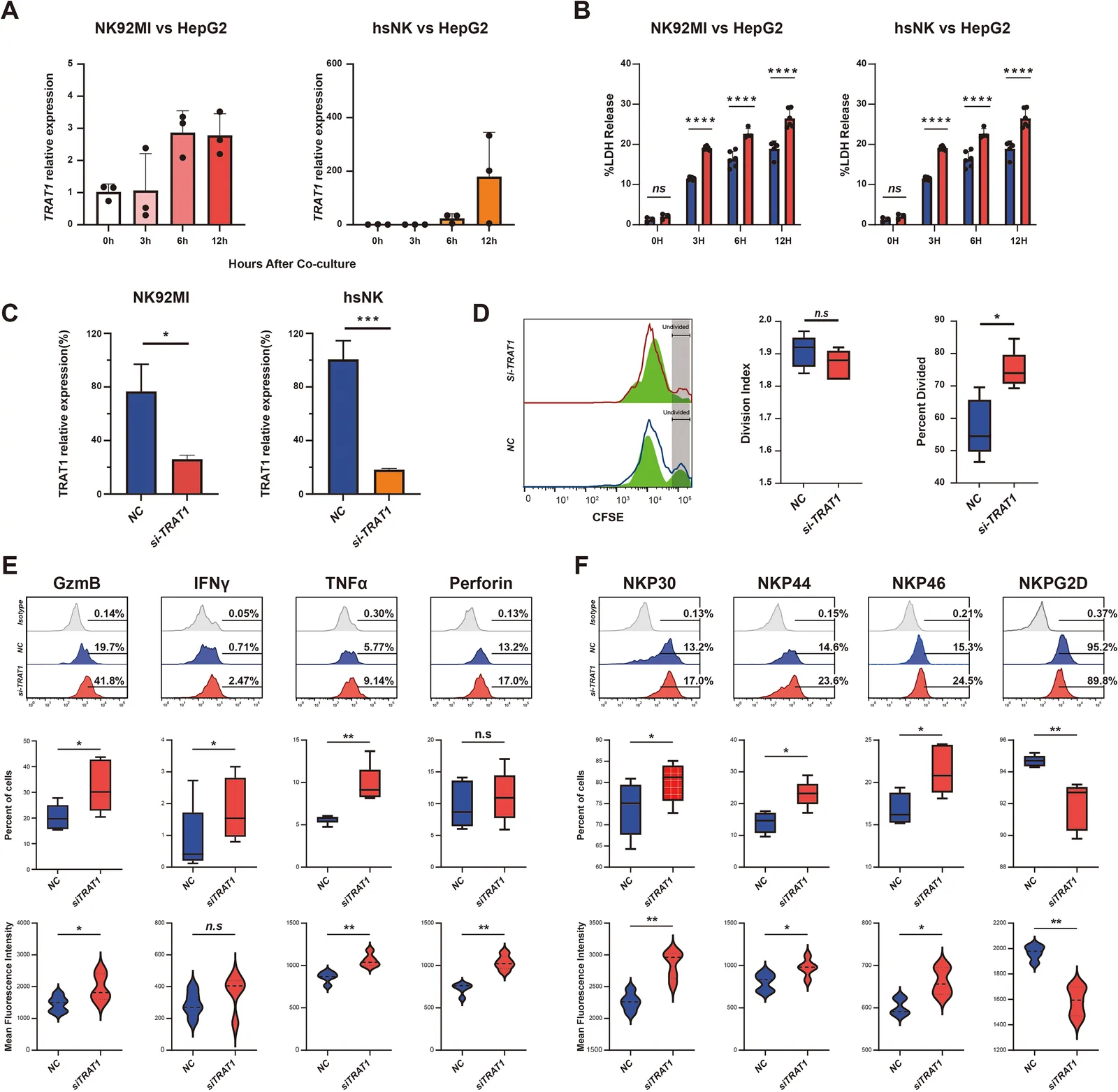
*TRAT1* modulates NK cell activation, proliferation, cytotoxicity, and receptor expression. **(A)** Time-course qPCR kinetics of TRAT1 transcripts following HepG2 co-culture (E:T ratio = 5:1) in human NK92-MI cells (left) and primary human NK cells (hsNK, right) at indicated time points. **(B)** Time-dependent HepG2 cytotoxicity profiles quantified via LDH release tracking in siRNA-treated NK92-MI (left) and primary cells (right) compared to negative controls. **(C)** Validation of siRNA-mediated TRAT1 knockdown efficiency in NK92-MI cells (left) and primary human NK cells (right) by qRT-PCR at 48 h post-nucleofection. Relative expression was calculated using the 0.5^ΔCt method with β-actin as the internal reference gene. **(D)** CFSE dilution proliferation profiles, division index, and the percentage of divided cells in NK92-MI cells after 48 hours of growth. **(E)** Intracellular cytokine production in TRAT1-KD NK92-MI cells. Representative flow cytometry histograms (top) show specific staining (colored peaks) overlaid with isotype controls (grey peaks) for GZMB, Perforin, IFN-γ, and TNF-α after 6 hours of PMA/Ionomycin stimulation. Quantification is presented as box plots (middle) for the percentage of positive cells and violin plots (bottom) for the Mean Fluorescence Intensity (MFI). TRAT1 KD significantly increased both the percentage and MFI of GZMB and TNF-α. Notably, IFN-γ showed an increased percentage but unchanged MFI, whereas Perforin exhibited significantly elevated MFI despite no significant change in the percentage of positive cells. **(F)** Surface expression of NK cell activation receptors on TRAT1-KD NK92-MI cells. Representative flow cytometry histograms (top) and quantification (bottom) of NCR1 (NKp46), NCR2 (NKp44), NCR3 (NKp30), and NKG2D expression on activated NC and si-TRAT1 treated NK cells. TRAT1 KD significantly increased NCR1 and NCR2 expression and significantly decreased NKG2D expression, while NCR3 remained unaffected. Data are representative of three independent experiments and are presented as mean ± SD unless otherwise indicated. Statistical significance was determined using an unpaired two-tailed Student’s t-test for single-time-point two-group comparisons, including primary human NK-cell assays. Time-course cytotoxicity data in panel **(b)** were analyzed using two-way ANOVA followed by Bonferroni correction for multiple comparisons. ns, not significant; *P < 0.05; **P < 0.01; ***P < 0.001; ****P < 0.0001. .

To elucidate the functional involvement of TRAT1 in NK cell biology, we used NK92-MI cells and enriched primary human NK (hsNK) cells as complementary *in vitro* systems. An *in vitro* activation system was established by co-culturing these effector cells with human HepG2 hepatoma cells at an effector-to-target (E:T) ratio of 5:1. Monitoring the transcriptional kinetics over a 12-hour timeline revealed that TRAT1 mRNA levels were progressively induced upon sustained target-cell engagement, peaking synchronously at 6 hours post-co-culture in both NK92-MI and primary human NK cells (**Figure 5A**).

To test the effects of this induction, we performed targeted gene silencing using small interfering RNA (siRNA). Standard lactate dehydrogenase (LDH) release assays demonstrated that TRAT1 knockdown increased HepG2 cytotoxicity in both NK92-MI cells and primary human NK cells across the measured time points (**Figure 5B**). Following these functional assays, transfection of specific si-TRAT1 resulted in a reduction of TRAT1 transcripts, as validated via real-time quantitative PCR (**Figure 5C**).

Given that our preliminary activation dynamics demonstrated a highly consistent expression profile and functional tendency between the NK92-MI cell line and primary human NK cells, and because NK92-MI cells provide a tractable system for reproducible transfection and multiparametric signaling assays, subsequent phenotypic and mechanistic analyses were performed primarily in this cell line. The primary human NK-cell experiments were used to assess whether the direction of the cytotoxicity phenotype was reproduced beyond the cell-line system. Functional analyses were then performed on TRAT1-KD NK cells. Cell proliferation was assessed by Carboxyfluorescein succinimidyl ester (CFSE) labeling; we observed that NK92-MI cells treated with si-TRAT1 exhibited significantly increased percent divided compared to the negative control (NC) group after 48 hours of spontaneous growth. These results suggest that TRAT1 KD enhances NK cell proliferation (**Figure 5D**). Together, these findings support a negative regulatory role for TRAT1 in NK cell-mediated cytotoxicity.

We further examined the effects of TRAT1 deficiency on key intracellular cytokines and surface activation markers in NK cells by flow cytometry. After stimulation with PMA/Ionomycin for 6 hours, NK cells with TRAT1 KD exhibited significantly elevated intracellular levels of IFN-γ, TNF-α, GZMB, and perforin. These increases likely account for the enhanced cytotoxicity observed in NK cells (**Figure 5E**). Furthermore, TRAT1 deficiency selectively altered the expression profile of NK-cell activation receptors. Specifically, the surface expression of NCR1 (NKp46) and NCR2 (NKp44) was upregulated in activated NK cells upon TRAT1 KD, as primarily evidenced by the increased mean fluorescence intensity (MFI) alongside an elevated frequency of positive cells. NCR3 (NKp30) expression remained largely unaffected. In contrast, NKG2D exhibited an opposite regulatory pattern, showing reduced expression (demonstrated by a marked decrease in MFI and positive cell percentage) after TRAT1 KD (**Figure 5F**).

### TRAT1 modulates calcium flux and MAPK signaling pathways in NK cell

**Figure 6:**
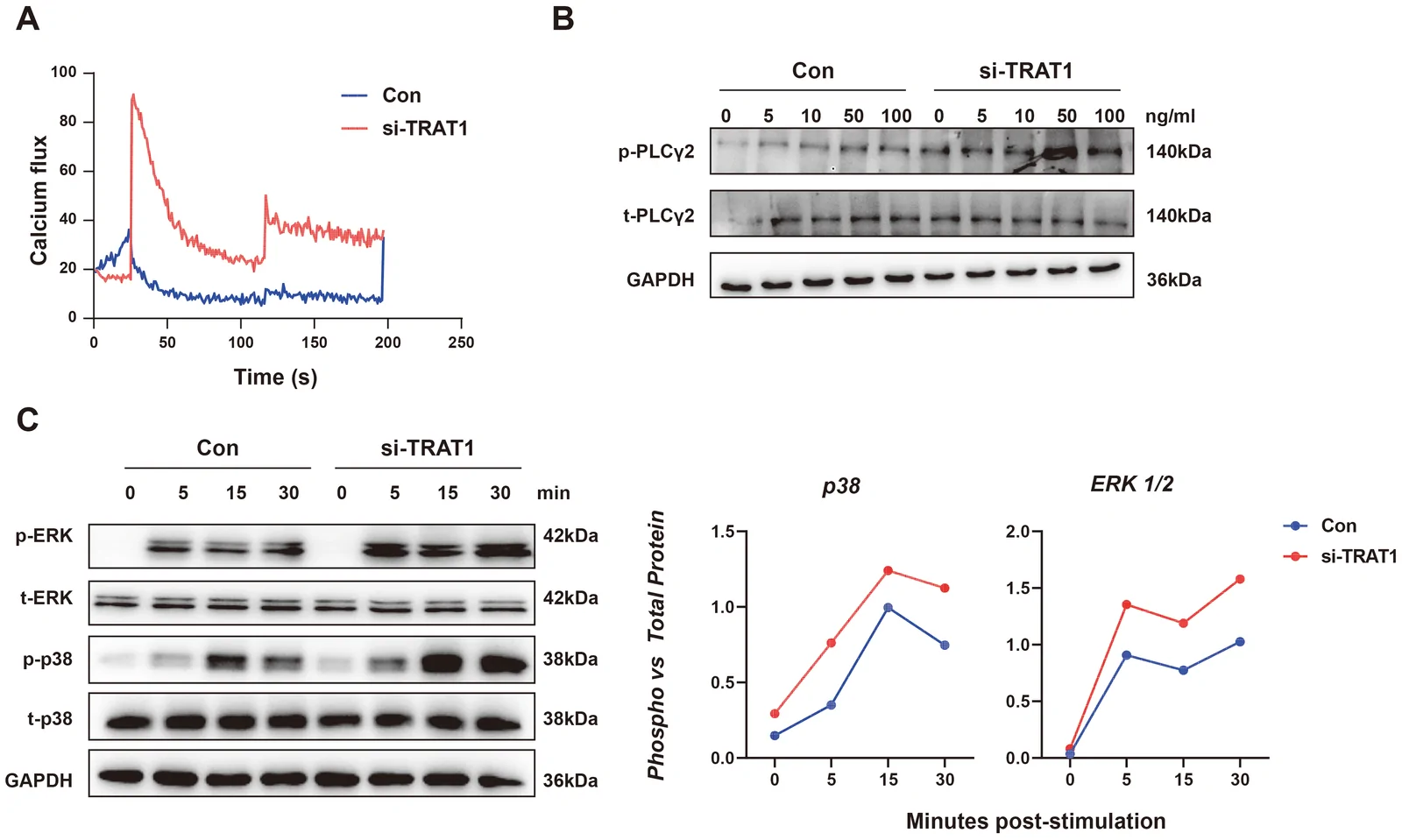
TRAT1 modulates calcium flux and MAPK signaling pathways in NK cells. **(A)** Calcium flux in control (Con) and TRAT1-KD (si-TRAT1) NK92-MI cells following stimulation with PMA/Ionomycin. TRAT1-KD NK cells exhibited significantly increased Ca²^+^influx over time. **(B)** Western blot analysis of phosphorylated PLCγ2 (p-PLCγ2) and total PLCγ2 (t-PLCγ2) in control (Con) and si-TRAT1 NK92-MI cells stimulated with varying concentrations of PMA/Ionomycin (0, 5, 10, 50, 100 ng/ml). Phosphorylation of PLCγ2 was markedly elevated in the absence of TRAT1. GAPDH was used as a loading control. **(c)** Western blot analysis (left) and quantification (right) of phosphorylated ERK1/2 (p-ERK), total ERK1/2 (t-ERK), phosphorylated p38 (p-p38), and total p38 (t-p38) in control (Con) and si-TRAT1 NK92-MI cells at different time points (0, 5, 15, 30 minutes) post-stimulation. TRAT1 KD resulted in markedly increased phosphorylation of ERK1/2 and p38. GAPDH was used as a loading control. Line graphs show the ratio of phosphorylated protein to total protein. Representative calcium-flux traces and immunoblots from three independent experiments are shown. The line graphs in panel **(C)** show the relative ratios of phosphorylated protein to total protein derived from the representative immunoblot. No inferential statistical analysis was applied to the representative data shown in this figure.

Calcium flux plays a critical role in NK cell activation; therefore, we assessed whether TRAT1 regulates calcium flux in NK cells. TRAT1- KD NK cells showed increased Ca²^+^influx following stimulation with PMA/Ionomycin (**Figure 6A**).

Consistent with this observation, phosphorylation of PLCγ2 (p-PLCγ2), a key mediator of calcium signaling, was also increased in the absence of TRAT1 (**Figure 6B**).

Further analysis of downstream signaling pathways revealed markedly increased phosphorylation of ERK1/2 and p38 in activated TRAT1- KD NK cells (**Figure 6C**), both kinases essential for NK cell activation and cytotoxicity. These results suggest that TRAT1 knockdown is associated with enhanced calcium flux and MAPK activation in NK92-MI cells, supporting its role as a candidate negative modulator of NK-cell effector function *in vitro*.

## Discussion

In this study, we utilized transcriptomic profiling of liver tissues to identify TRAT1 as a candidate immune-related gene associated with AIH. Through an integrative approach, we subsequently demonstrated that Trat1 is selectively upregulated in hepatic NK cells within a murine model of autoimmune liver injury. Functional characterization in NK92-MI cells showed that TRAT1 knockdown was associated with an augmented effector phenotype, characterized by increased proliferation, enhanced cytotoxicity, and the upregulation of key effector molecules (GZMB, TNF-α) and specific natural cytotoxicity receptors (NCRs) such as NKp44 and NKp46. These findings extend the known functional repertoire of TRAT1 beyond T-cell biology and suggest it acts as a negative regulator of NK cell responses in an *in vitro* context.

Our initial transcriptomic analysis of liver tissues from AIH patients compared to PBC and CHB controls identified a set of IRDEGs, with Gene Ontology analysis pointing towards dysregulation in lymphocyte-mediated immunity, T-cell receptor complex signaling, and antigen binding. Among these, eight genes, including TRAT1, showed significantly higher expression in AIH livers and demonstrated exploratory discriminatory potential. This initial finding highlighted TRAT1 as a gene of interest in the context of AIH. To further contextualize these findings and explore the relevance of TRAT1 in an AIH context, we utilized public datasets. In a Concanavalin A (ConA)-induced acute immune-mediated hepatitis model, Trat1 (the murine ortholog) emerged as a key prioritized candidate gene through an integrative analysis of DEGs and WGCNA co-expression modules. This model also revealed alterations in several immune cell populations, including activated NK cells. This strategy of cross-referencing clinical tissue transcriptomics with public datasets aligns with the expanding paradigm of utilizing integrative multi-omics pipelines to decode hepatobiliary-specific immune gene patterns and prioritize functional candidates in gastrointestinal and hepatic inflammatory diseases (11).

To examine the cellular distribution of Trat1 *in vivo*, we used a Concanavalin A (ConA)-induced acute immune-mediated hepatitis model. This model produced acute hepatocyte injury characterized by inflammatory infiltration and elevated serum ALT. Flow cytometric analysis of hepatic mononuclear cells revealed altered proportions of intrahepatic lymphocyte subsets, and qPCR on FACS-sorted populations showed that Trat1 expression was elevated in the hepatic NK-cell-enriched compartment, while remaining largely unchanged in helper T and cytotoxic T cell compartments. A similar trend of NK-restricted Trat1 elevation was also observed in a preliminary CYP2D6 plasmid-induced chronic immune-mediated liver injury model (**Supplementary Figure 1**), providing additional supportive context. Together, these *in vivo* observations align with the bulk transcriptomic finding in human AIH liver tissue and point to a potential role for TRAT1 in NK cells within the inflamed liver.

Natural killer (NK) cells serve as critical effectors in immune-mediated diseases, whose activation kinetics, cytotoxicity, and cytokine output are precisely governed by intricate intracellular signaling adaptors and networks (12). Given the upregulation of TRAT1 in hepatic NK cells in the AIH model, we investigated its functional role in NK cell biology using the NK92-MI cell line, where TRAT1 mRNA expression was transiently upregulated upon target cell co-culture. siRNA-mediated KD of TRAT1 revealed its significant negative regulatory role in NK cell effector functions, leading to enhanced proliferation, increased cytotoxicity, and elevated production of IFN-γ, TNF-α, GZMB, and perforin. These findings suggest that TRAT1 acts as a negative regulator dampening NK cell response.

Interestingly, while TRAT1 KD augmented the expression of NKp44 and NKp46, it resulted in the downregulation of NKG2D. The precise mechanism behind this specific downregulation remains unclear. One possible explanation is that NKG2D is downregulated as a compensatory negative feedback mechanism in response to the enhanced activation state of TRAT1-deficient NK cells, or that TRAT1 plays a distinct structural role in stabilizing surface NKG2D expression. This novel observation warrants further mechanistic investigation (13, 14). Our study found TRAT1 KD significantly augmented Ca²^+^influx, PLCγ2 phosphorylation, and phosphorylation of ERK1/2 and p38. These results suggest TRAT1 dampens NK cell function by limiting calcium flux and subsequent MAPK pathway activation, acting as a negative regulator.

The upregulation of TRAT1 in hepatic NK cells in our AIH model, combined with its negative regulatory role on NK cell effector functions, suggests a complex scenario. In AIH, the increased TRAT1 in liver NK cells might be a counter-regulatory attempt to limit excessive NK cell-mediated hepatocyte damage. However, insufficient upregulation or overriding factors could still allow NK cells to contribute to liver pathology, or sustained high TRAT1 expression might lead to NK cell dysfunction.

It is also noteworthy that the functional role of TRAT1 appears to be highly context dependent. A recent study by Xiao et al. reported that TRAT1 functions as a tumor suppressor in lung adenocarcinoma, where its expression is downregulated in tumor tissues, and its overexpression inhibits cancer cell proliferation and migration (8). This contrasts with our findings in AIH, where TRAT1 is upregulated in hepatic NK cells and acts to dampen cytotoxic effector functions. This dichotomy suggests that TRAT1 plays distinct roles depending on the pathological microenvironment: in oncology, its loss may promote tumor progression, whereas in autoimmunity, its upregulation likely serves as a negative feedback mechanism to limit excessive immune-mediated tissue damage. Further studies are required to determine whether the context-dependent functions of TRAT1 have translational relevance.

To clarify the methodological boundaries of this study, it is essential to delineate the distinct and non-interchangeable roles of the four data layers integrated within our workflow. First, our in-house human bulk RNA-seq cohort serves strictly as the clinical discovery anchor, comparing AIH liver tissues against relevant disease controls (PBC and CHB). Second, the public murine dataset (GSE45413) represents a concanavalin A (ConA)-induced acute immune-mediated hepatitis model; we utilize this layer not as a complete pathological equivalent to chronic human AIH, but rather as a tractable system to capture acute leukocyte activation and hepatic co-expression changes. Third, the public human scRNA-seq dataset (GSE216064) reflects peripheral blood mononuclear cells (PBMCs), providing a baseline descriptive snapshot of circulating immune cells, which fundamentally differs from the tissue-resident microenvironment of the liver. Fourth, our in-house ConA-induced acute immune-mediated hepatitis model serves solely to execute *in vivo* functional and cell-type specific qPCR validations. These data layers are treated as complementary rather than interchangeable, and our conclusions are carefully restricted within these methodological boundaries.

In response to the preliminary nature of the PBMC scRNA-seq analysis, we further examined TRAT1 transcript distribution across annotated immune-cell subsets and performed an exploratory TRAT1-centered analysis within NK cells from the AIH sample. This analysis showed that TRAT1 transcripts were detectable in lymphoid compartments, including NK cells. However, no clear transcript-level correlation was observed between TRAT1 and Ca²^+^/PLCγ2/MAPK-related module scores in peripheral NK cells. This lack of transcript-level correlation should be interpreted cautiously and does not necessarily conflict with the NK92-MI mechanistic data. Ca²^+^influx is a dynamic functional event, whereas PLCγ2, ERK1/2, and p38 activation is primarily determined by phosphorylation rather than steady-state mRNA abundance. The intrinsic transcript dropout and zero-inflation of single-cell data further limit the detectability of any transcript-level correlation. Moreover, this public dataset contains only one AIH sample and one healthy-control sample and represents peripheral blood rather than liver tissue. Therefore, the single-cell analysis provides peripheral transcriptomic context but cannot substitute for functional signaling assays. The mechanistic link between TRAT1 knockdown and enhanced Ca²^+^/PLCγ2/MAPK signaling remains supported primarily by the NK92-MI experiments and requires future validation in primary NK cells and liver-derived AIH samples.

While our study provides valuable exploratory insights into the role of TRAT1, we acknowledge several important limitations. First, the human bulk RNA-seq dataset utilized a small cohort (n=3 per group) without healthy liver controls, and the human scRNA-seq validation was limited to a single AIH patient, warranting caution. Given the inherent technological noise and sequencing benchmarking limits often encountered when characterizing dynamic leukocyte lineages via single-cell transcriptomic algorithms (15), we have strictly confined this data layer to a qualitative and descriptive overview of peripheral cell-type tendencies. Consequently, while our murine model demonstrated robust TRAT1 upregulation specifically in hepatic NK cells, the direct translation of this cell-type-specific pattern to human AIH requires definitive validation in larger clinical cohorts. Second, although the NK-92MI cell line provided a tractable and reproducible model for our mechanistic assays, it cannot perfectly capture the complex phenotypic heterogeneity and microenvironmental interactions of primary human tissue-resident NK cells. Although we extended TRAT1 expression and knockdown-mediated cytotoxicity assessments to primary human NK cells from healthy donors, the downstream phenotypic and signaling characterizations (proliferation, intracellular cytokines, surface receptor profiling, Ca²^+^flux, and MAPK signaling) were performed exclusively in NK92-MI cells. Future studies are needed to validate these downstream readouts in primary NK cells, ideally those isolated from AIH patients. Third, we did not evaluate the overall therapeutic efficacy of targeting TRAT1 in the AIH mouse model. Because TRAT1 plays established and potentially distinct roles in T cells versus NK cells, utilizing global interventions in this complex *in vivo* system is challenging. This restricts our current ability to evaluate the systemic safety of TRAT1 modulation, and we plan to use cell-specific conditional knockout mice for further verification in further work. Finally, the precise mechanisms driving TRAT1 upregulation in hepatic NK cells during AIH, as well as its direct physical interactions with upstream signaling components, need further investigation. The modest absolute frequency of cytokine-positive NK92-MI cells following PMA/ionomycin stimulation, despite robust target-cell killing, reflects intrinsic features of this readout system. NK92-MI is engineered for autocrine IL-2 signaling and maintains an elevated basal effector state, narrowing the dynamic range available for further pharmacological induction; in addition, intracellular cytokine staining captures only protein retained during a restricted Brefeldin A window and is not directly comparable to cumulative LDH-based cytotoxicity. Under matched stimulation and gating conditions, TRAT1 knockdown consistently increased the frequency of effector-molecule-positive cells, indicating a relative enhancement of NK92-MI activation.

In conclusion, our integrative transcriptomic analysis prioritized TRAT1 as a candidate gene of interest in AIH-associated NK cell biology. The exploratory functional assays in NK92-MI cells, supplemented by parallel assessments in primary human NK cells, suggest that TRAT1 is an *in vitro* candidate negative modulator of NK-cell effector responses, potentially involving calcium and MAPK signaling in NK92-MI cells. Given the inherent limitations of the sample size and experimental systems utilized herein, these findings provide a mechanistic lead that requires further validation in larger clinical cohorts, primary tissue-resident cells, and lineage-specific conditional knockout *in vivo* models.

## Data Availability

The datasets presented in this study can be found in online repositories. The names of the repository/repositories and accession number(s) can be found below: https://www.ncbi.nlm.nih.gov/geo/, GSE304352.

## Supplementary material

Supplementary Figure 1 Trat1 expression in hepatic immune cells of a CYP2D6 plasmid-induced chronic immune-mediated liver injury model. **(A)** Representative H&E-stained liver sections from healthy control (HC) mice and mice with AIH induced by CYP2D6 plasmid injection. AIH model mice exhibit a marked inflammatory phenotype. The red boxes indicate the regions selected for higher magnification. High-magnification insets (top right) show detailed inflammatory infiltration. Scale bars = 100 μm **(B)** Serum alanine aminotransferase (ALT) and aspartate aminotransferase (AST) levels in HC and AIH model mice. AIH mice show significantly elevated ALT levels. Data are presented as mean ± SEM or individual points with mean. ns, not significant; *P < 0.05; **P < 0.01; ***P < 0.001; ****P < 0.0001. **(C)** Flow cytometric analysis of hepatic lymphocyte populations in HC and AIH model mice. Representative flow cytometry plots (left) show gating strategy for Helper T cells (Th; CD4+CD8-), cytotoxic T cells (Tc; CD4-CD8+), NK cells (NK1.1+B220-CD4-CD8-), and B lymphocytes (B220+NK1.1-CD4-CD8-). Bar graphs (right) show the percentage of these lymphocyte subsets within total liver lymphocytes. AIH model mice show increased proportions of Th, Tc, NK, and B cells, with the most substantial rise in T cell populations and an elevation trend for NK cells. *P < 0.05. **(D)** Relative *TRAT1* gene expression in sorted hepatic Th cells, Tc cells, NK cells, and B cells from HC and AIH model mice, assessed by qPCR. *TRAT1* expression is significantly enhanced solely in the NK-cell-enriched population from AIH model mice. Data are representative of at least two independent experiments and are presented as mean ± SD (n=8 mice per group). Statistical significance between the healthy-control and CYP2D6-injected (AIH model) groups was determined using an unpaired two-tailed Student’s t-test for each indicated comparison. ns, not significant; *P < 0.05; **P < 0.01; ***P < 0.001; ****P < 0.0001.

Supplementary Figure 2 Exploratory TRAT1-centered transcript-level correlation analysis in NK cells from the public AIH PBMC scRNA-seq dataset. Left: correlation matrix showing transcript-level correlations among TRAT1 and selected Ca²^+^/PLCγ2/MAPK-related genes in NK cells from the AIH sample. Right: scatter plots showing the relationship between TRAT1 expression and Ca²^+^signaling, PLCγ2-related, ERK/MAPK, and p38/MAPK transcript-level module scores in NK cells. Values above each scatter plot indicate Spearman correlation coefficients. No clear transcript-level correlation was observed between TRAT1 and these signaling-related transcript scores. Because the dataset contains one AIH sample and one healthy-control sample, these analyses are presented descriptively without inferential statistical testing. These transcript-level analyses do not directly measure Ca²^+^influx or phosphorylation-dependent activation of PLCγ2, ERK1/2, or p38.
