# Correction: Transcriptomic profiling of autoimmune hepatitis identifies TRAT1 as an *in vitro* negative regulator of NK cell effector functions

**DOI:** 10.3389/fimmu.2026.1932984

**Published:** 2026-08-07

**Authors:** Jiapeng Gao, Lixia Huo, Jianfeng Zhong, Jiexun Cai, Jing Yu, Jingwen Li, Junbo Jiang, Chuanzi Hong, Yunliang Yao, Min Feng

**Affiliations:** 1 First Affiliated Hospital, Huzhou Normal University, Huzhou, China; 2 School of Medicine & Nursing, Huzhou Normal University, Huzhou, China; 3 Huzhou Central Hospital, The Affiliated Central Hospital of Huzhou Normal University, Huzhou, China

**Keywords:** AIH (autoimmune hepatitis), negative regulator, NK cell, PLCγ/Ca2+ pathway, TRAT1

There was a mistake in **Figure 6** as published. In **Figure 6A**, the red data curve is completely missing. In **Figure 6C**, the connecting lines between the red data points in the line graphs (p38 and ERK 1/2) are missing.

**Figure 6:**
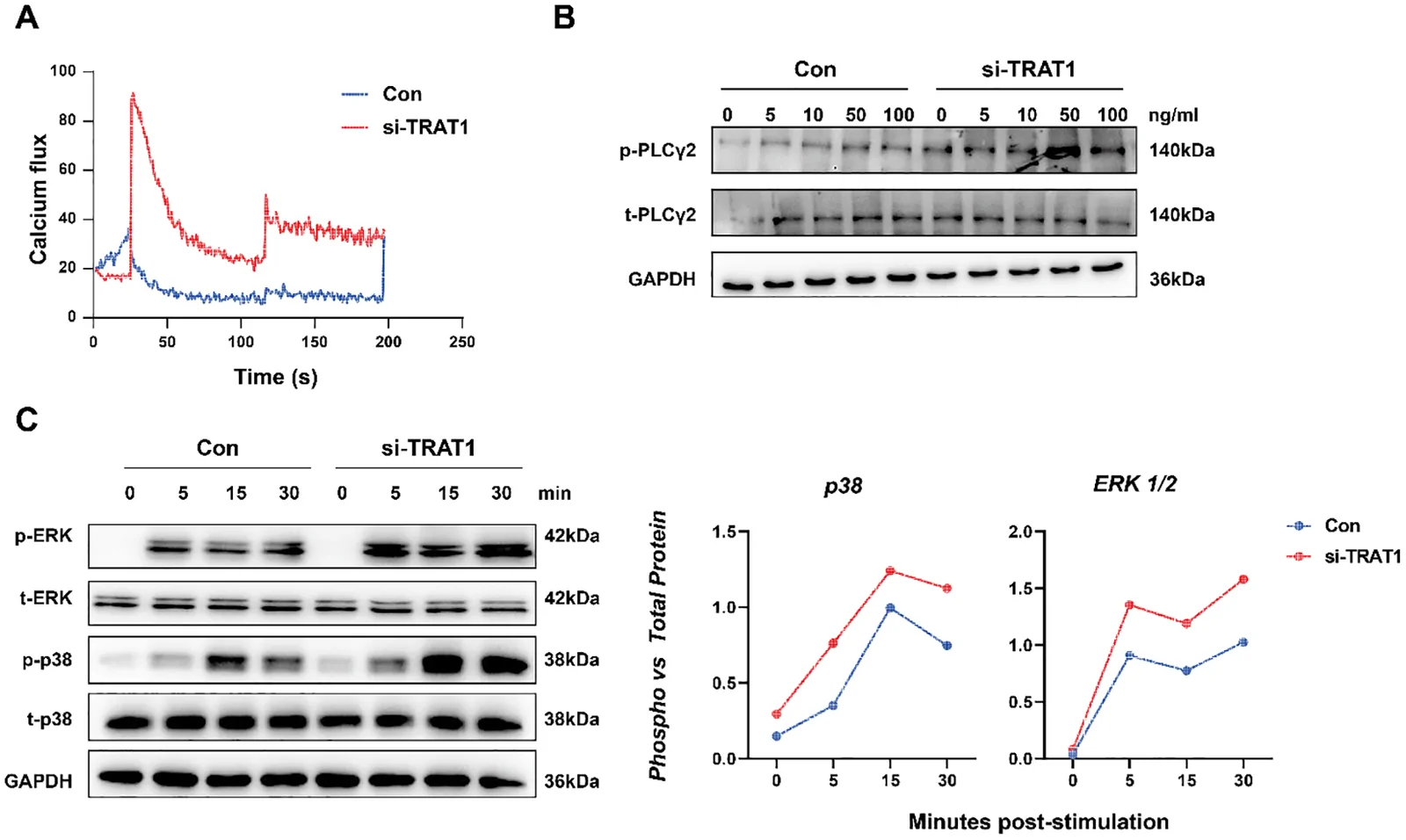
TRAT1 modulates calcium flux and MAPK signaling pathways in NK cells. **(A)** Calcium flux in control (Con) and TRAT1-KD (si-TRAT1) NK92-MI cells following stimulation with PMA/Ionomycin. TRAT1-KD NK cells exhibited significantly increased Ca²+influx over time. **(B)** Western blot analysis of phosphorylated PLCγ2 (p-PLCγ2) and total PLCγ2 (t-PLCγ2) in control (Con) and si-TRAT1 NK92-MI cells stimulated with varying concentrations of PMA/Ionomycin (0, 5, 10, 50, 100 ng/ml). Phosphorylation of PLCγ2 was markedly elevated in the absence of TRAT1. GAPDH was used as a loading control. (c) Western blot analysis (left) and quantification (right) of phosphorylated ERK1/2 (p-ERK), total ERK1/2 (t-ERK), phosphorylated p38 (p-p38), and total p38 (t-p38) in control (Con) and si-TRAT1 NK92-MI cells at different time points (0, 5, 15, 30 minutes) post-stimulation. TRAT1 KD resulted in markedly increased phosphorylation of ERK1/2 and p38. GAPDH was used as a loading control. Line graphs show the ratio of phosphorylated protein to total protein. Representative calcium-flux traces and immunoblots from three independent experiments are shown. The line graphs in panel **(C)** show the relative ratios of phosphorylated protein to total protein derived from the representative immunoblot. No inferential statistical analysis was applied to the representative data shown in this figure..

The corrected **Figure 6** appears below.

The original version of this article has been updated.

