# Supplementary figures and images for: Transcriptomic profiling of autoimmune hepatitis identifies TRAT1 as an in vitro negative regulator of NK cell effector functions
Source: Front Immunol. 2026 Jul 7;17:1843865. doi: 10.3389/fimmu.2026.1843865 (PMC13384938; doi:10.3389/fimmu.2026.1843865)

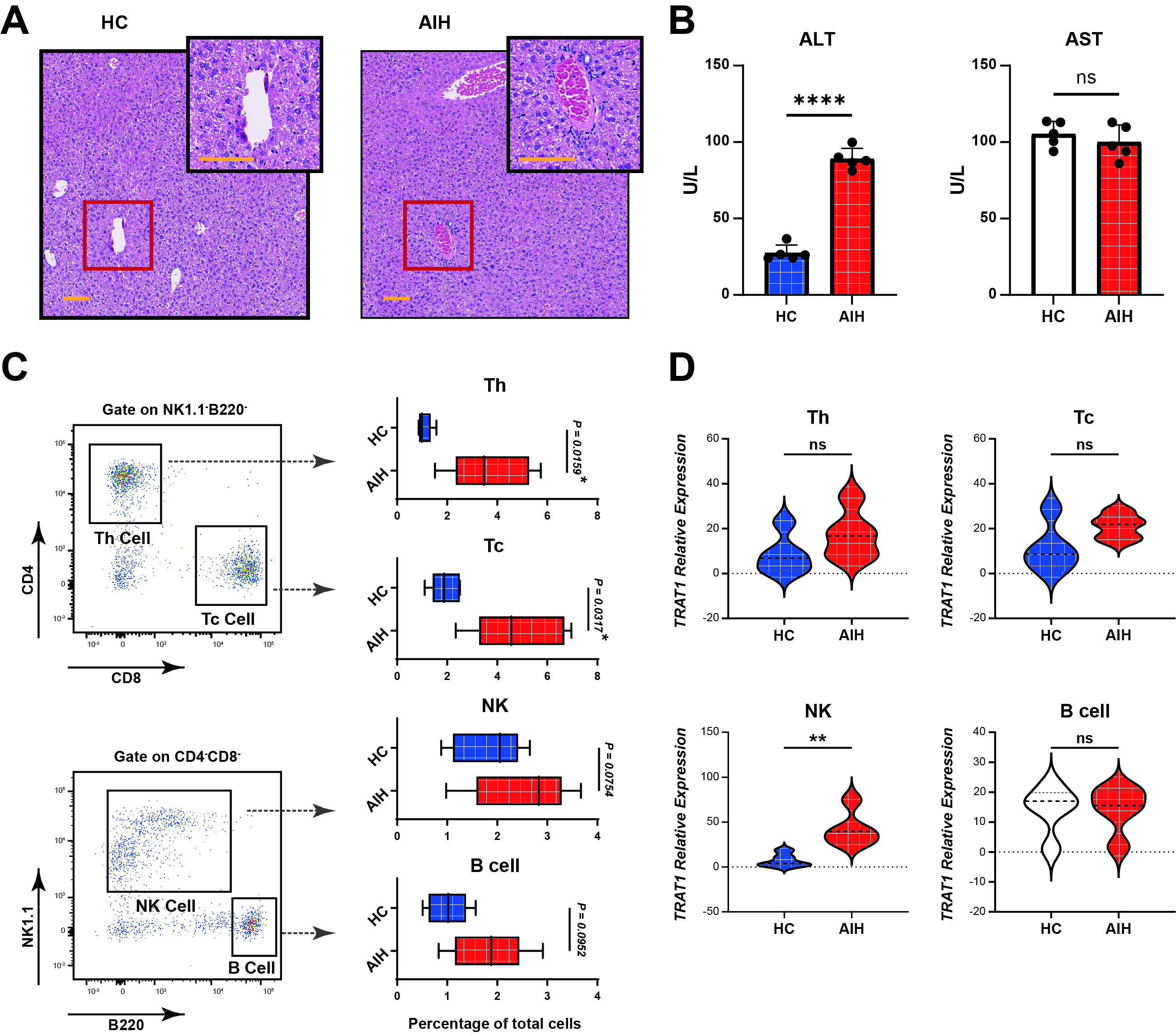

Supplement: Supplementary Figure 1 — Trat1 expression in hepatic immune cells of a CYP2D6 plasmid-induced chronic immune-mediated liver injury model. (A) Representative H&E-stained liver sections from healthy control (HC) mice and mice with AIH induced by CYP2D6 plasmid injection. AIH model mice exhibit a marked inflammatory phenotype. The red boxes indicate the regions selected for higher magnification. High-magnification insets (top right) show detailed inflammatory infiltration. Scale bars = 100 μm (B) Serum alanine aminotransferase (ALT) and aspartate aminotransferase (AST) levels in HC and AIH model mice. AIH mice show significantly elevated ALT levels. Data are presented as mean ± SEM or individual points with mean. ns, not significant; *P < 0.05; **P < 0.01; ***P < 0.001; ****P < 0.0001. (C) Flow cytometric analysis of hepatic lymphocyte populations in HC and AIH model mice. Representative flow cytometry plots (left) show gating strategy for Helper T cells (Th; CD4+CD8-), cytotoxic T cells (Tc; CD4-CD8+), NK cells (NK1.1+B220-CD4-CD8-), and B lymphocytes (B220+NK1.1-CD4-CD8-). Bar graphs (right) show the percentage of these lymphocyte subsets within total liver lymphocytes. AIH model mice show increased proportions of Th, Tc, NK, and B cells, with the most substantial rise in T cell populations and an elevation trend for NK cells. *P < 0.05. (D) Relative TRAT1 gene expression in sorted hepatic Th cells, Tc cells, NK cells, and B cells from HC and AIH model mice, assessed by qPCR. TRAT1 expression is significantly enhanced solely in the NK-cell-enriched population from AIH model mice. Data are representative of at least two independent experiments and are presented as mean ± SD (n=8 mice per group). Statistical significance between the healthy-control and CYP2D6-injected (AIH model) groups was determined using an unpaired two-tailed Student’s t-test for each indicated comparison. ns, not significant; *P < 0.05; **P < 0.01; ***P < 0.001; ****P < 0.0001. [file Image1.jpeg]

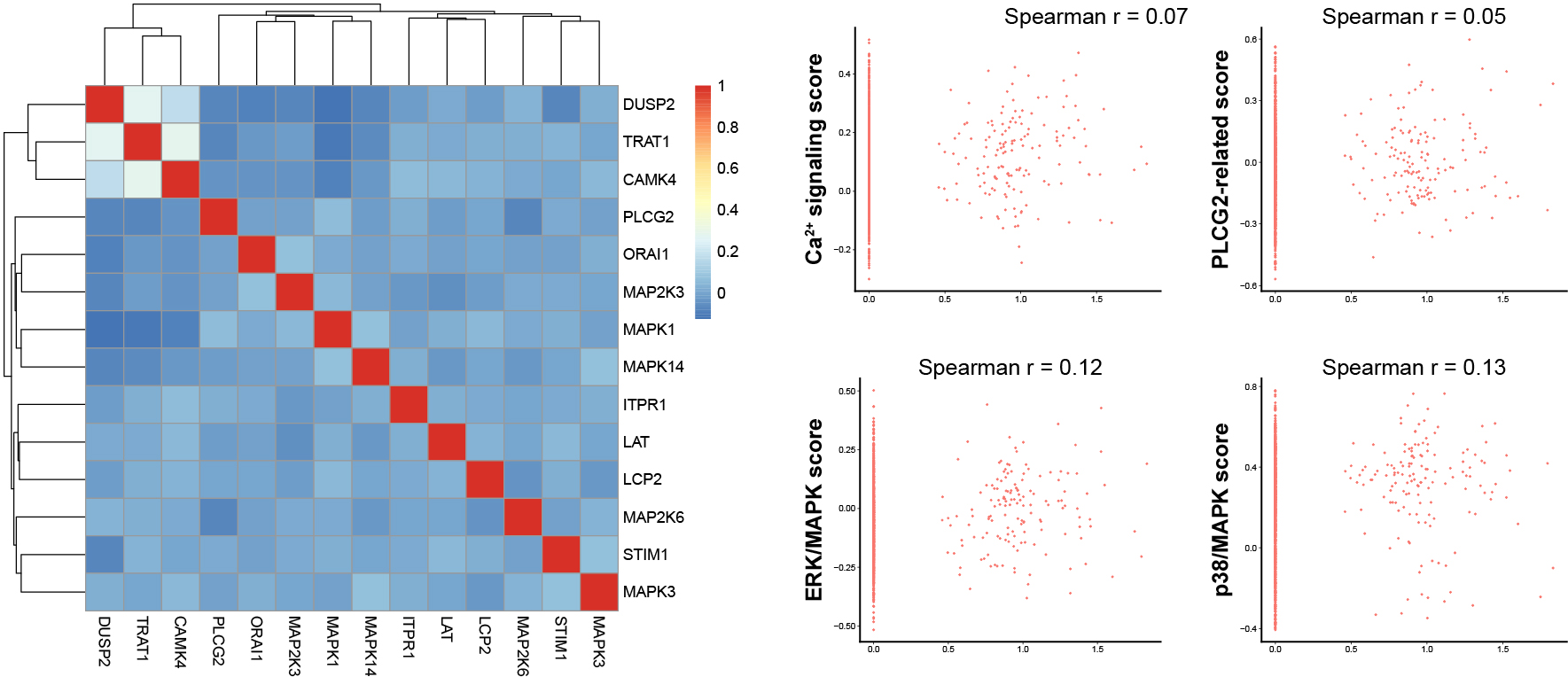

Supplement: Supplementary Figure 2 — Exploratory TRAT1-centered transcript-level correlation analysis in NK cells from the public AIH PBMC scRNA-seq dataset. Left: correlation matrix showing transcript-level correlations among TRAT1 and selected Ca²+/PLCγ2/MAPK-related genes in NK cells from the AIH sample. Right: scatter plots showing the relationship between TRAT1 expression and Ca²+signaling, PLCγ2-related, ERK/MAPK, and p38/MAPK transcript-level module scores in NK cells. Values above each scatter plot indicate Spearman correlation coefficients. No clear transcript-level correlation was observed between TRAT1 and these signaling-related transcript scores. Because the dataset contains one AIH sample and one healthy-control sample, these analyses are presented descriptively without inferential statistical testing. These transcript-level analyses do not directly measure Ca²+influx or phosphorylation-dependent activation of PLCγ2, ERK1/2, or p38. [file Image2.jpeg]
